# Widespread occurrence of fecal indicator bacteria in oligotrophic tropical streams. Are common culture-based coliform tests appropriate?

**DOI:** 10.7717/peerj.18007

**Published:** 2024-09-06

**Authors:** Karina Chavarria, Jorge Batista, Kristin Saltonstall

**Affiliations:** 1 Smithsonian Tropical Research Institute, Panama City, Panama; 2 Department of Civil and Environmental Engineering, University of Massachusetts at Amherst, Amherst, MA, United States of America

**Keywords:** Culture-based indicator bacteria, Oligotrophic streams, Tropical watersheds, 16S metabarcoding, Colilert, Water quality monitoring

## Abstract

Monitoring of stream water quality is a key element of water resource management worldwide, but methods that are commonly used in temperate habitats may not be appropriate in humid tropical systems. We assessed the influence of four land uses on microbial water quality in 21 streams in the Panama Canal Watershed over a one-year period, using a common culture-based fecal indicator test and 16S rDNA metabarcoding. Each stream was located within one of four land uses: mature forest, secondary forest, silvopasture, and traditional cattle pasture. Culturing detected total coliforms and *Escherichia coli* across all sites but found no significant differences in concentrations between land uses. However, 16S rDNA metabarcoding revealed variability in the abundance of coliforms across land uses and several genera that can cause false positives in culture-based tests. Our results indicate that culture-based fecal indicator bacteria tests targeting coliforms may be poor indicators of fecal contamination in Neotropical oligotrophic streams and suggest that tests targeting members of the Bacteroidales would provide a more reliable indication of fecal contamination.

## Introduction

Surface waters are particularly vulnerable to contamination, unlike many groundwater sources that have natural protection provided by overlying soils. Fecal contamination is typically monitored by testing for fecal indicator bacteria (FIB), particularly when pathogens of interest are not easily measured or cultured (Martin et al., 2013). FIB, which include total coliforms, fecal coliforms, *Escherischia coli*, fecal streptococci, and enterococci, have been widely accepted as a measure of water safety since the late 19th century (Hazen, 1988), and their monitoring through the use of culture-based approaches, in combination with traditional water quality parameters (*e.g.*, turbidity, dissolved oxygen, *etc.*), has become the standard for assessing water quality (Baxter-Potter & Gilliland, 1988; Davies-Colley et al., 2004; Hazen & Toranzos, 1990; Kumpel & Nelson, 2014). However, there is a concern that commonly used culture-based tests that depend on selective media may not adequately measure fecal contamination due to the high prevalence of other bacteria (*e.g.*, *Pseudomonas spp*.) that can generate false positives and that the presence of these organisms in tropical freshwater environments may not represent fecal contamination (Viau et al., 2011). Thus, understanding the effectiveness of common culture-based tests for the detection of fecal pollution in tropical water environments can help to better characterize risks, especially public health risks of water-borne and zoonotic diseases, and improve strategies to protect water quality.

This study assessed microbial water quality in oligotrophic tropical streams influenced by four common land uses in the Neotropics using the common culture-based test Colilert^®^ (IDEXX Laboratories Inc., Westbrook, ME, USA), 16S metabarcoding and traditional physicochemical parameters. Colilert^®^ is a widely used indicator test that uses enzyme-based media to quantify total coliform bacteria and *Escherichia coli* simultaneously. We hypothesized that streams influenced by cattle pastures would have a higher prevalence of FIB than protected forested streams. Our main objectives were to: (1) assess differences in the concentrations of indicator bacteria in streams influenced by different land uses using Colilert^®^; (2) use culture-independent 16S rDNA metabarcoding to describe the bacterial assemblages in these streams, with a focus on coliform bacteria; and (3) compare culture-based Colilert^®^ and 16S metabarcoding results and investigate the capability of these approaches.

## Materials & Methods

### Study site

**Figure 1:**
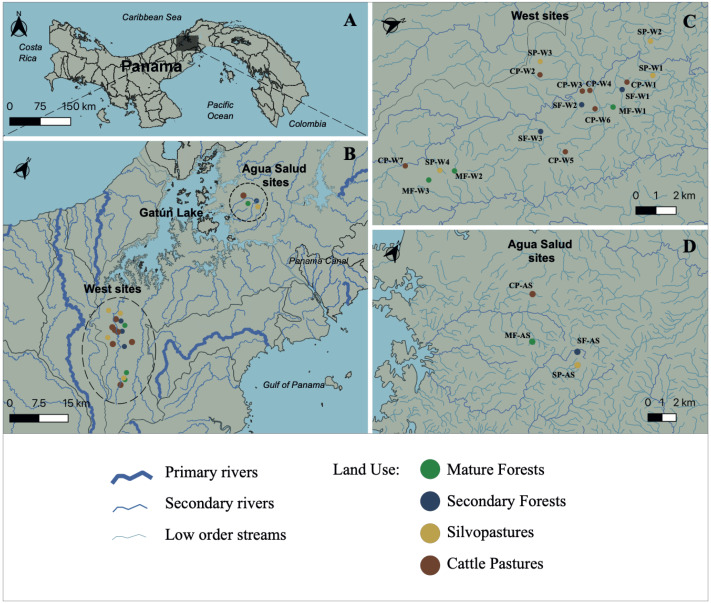
Study site. Map of Panama indicating (A) the primary area of study; (B) the Panama Canal Watershed and the sampling locations; (C) Sampling locations on the western side of the Panama Canal Watershed; and (D) Agua Salud sampling locations. Watershed and stream geographical data was sourced from STRI’s ArcGIS open-data program (*Physical Monitoring Program of the Smithsonian Tropical Research Institute*; Paton, 2020). MF, Mature Forest (*n* = 4 sites); SF, Secondary Forest (*n* = 4 sites); SP, Silvopasture (*n* = 5 sites); CP, Cattle Pasture (*n* = 8 sites). Map was created using the open source geographic information system QGIS-LTR (Version 3.22.8).

This study was carried out at 21 streams in the Panama Canal Watershed (PWC), Panama, for one year during 2018 to 2019 (Figs. 1A–1D). Water samples were collected from 17 streams in the western part of the Panama Canal watershed (Fig. 1C) and in four experimental watersheds within the Agua Salud facility of the Smithsonian Tropical Research Institute (STRI) in the eastern Panama Canal watershed (Fig. 1D). Each stream was bordered by one of four land uses that are common in the Neotropics: (1) mature forests (>80 years in age (MF)); (2) secondary forests (<25 years in age (SF)); (3) silvopastures (SP) and; (4) traditional cattle pastures (CP). Silvopastures differ from traditional cattle pastures in that the stream channels are bordered by riparian forest, streams are fenced to allow cattle drinking water access at only one point, and more trees are left in the pastures. All streams were specifically selected because their adjacent land use was not confounded by other upstream uses. Landowners granted permission to sample water on their properties. This research had sampling permission from the Republic of Panama Ministry of Environment by permits (SE/PO-2-19) and (SE/AP-2-18).

### Sampling and physicochemical measurements

Biweekly water samples and physicochemcial measurements were collected from each stream following protocols described in Chavarria et al. (2021), Murphy & Stallard (2012) and Paton (2020). Most samples were collected between 8:00 A.M. and 12:00 P.M. during baseflow conditions, and all samples were transported at 4 °C to STRI’s laboratory for processing within three hours of collection (Chavarria, 2021).

Following Chavarria (2021), turbidity, pH, temperature, conductivity, total dissolved solids and dissolved oxygen were measured and recorded on site during sample collection. Turbidity was measured with a field-portable turbidimeter (MicroTPW, HF Scientific Inc., Fort Myers, FL, USA). Temperature, pH, conductivity and total dissolved solids were measured with a HANNA portable multiparameter meter (HANNA Instruments, Smithfield, RI, USA) and dissolved oxygen was measured using a portable dissolved oxygen meter (HANNA Instruments, Smithfield, RI, USA), following manufacturer’s recommendations.

Total coliform and *E. coli* concentrations were determined from 100 mL water samples using the IDEXX Colilert^®^/Quanti-tray 2000 culture assay, following the manufacturer’s recommendations (IDEXX Laboratories, Inc., Westbrook, ME, USA) (Chavarria, 2021). Samples were incubated at approximately 35 °C for 24 h and positive wells were counted. The IDEXX Quanti-Tray^®^/2000 MPN Table was used to convert the number of positive wells to the most probable number (MPN). Microbial detection limits for total coliform and *E. coli* had a lower bound of <1 MPN/100 mL and an upper bound of >2419.6 MPN/100 mL (Chavarria, 2021). Field and laboratory positive (internal pure-culture samples) and negative controls (consisting of autoclaved water exposed to the field and laboratory environment) were measured periodically during sampling, and contamination was not detected.

16S rDNA metabarcoding was also used to assess the presence of coliforms and *E. coli* in the total bacterial community. Water samples (1L) were collected and processed according to protocols described in Chavarria et al. (2021). Briefly, the water was filtered in a sterile fashion using a vacuum pump filtration system (Laramie et al., 2015) and DNA was isolated from 0.22 µm mixed cellulose filters (EDM Millipore, Burlington, MA, USA) using the DNeasy PowerSoil Kit (QIAGEN, Hilden, Germany). The V4 region of the 16S rRNA gene was amplified in triplicate 12.5 uL reactions using a two-step PCR protocol using the phased primers 515F (Parada, Needham, & Fuhrman, 2016) and 806R (Apprill, Parsons & Weber, 2015) (Caporaso et al., 2011). Negative and positive controls containing nuclease-free water and previously PCR-positive samples were included in all PCR amplification steps. After a second PCR to add Illumina adaptors and sample specific barcodes, products were cleaned and normalized using normalization plates (Charm Biotech, San Diego, CA, USA) and pooled into two sequencing libraries (Chavarria, 2021). The libraries were further concentrated using magnetic beads, quantified by Qubit High Sensitivity dsDNA Assay (Thermo Fisher Scientific, Waltham, MA, USA), and quality checked by a Bioanalyzer dsDNA High Sensitivity assay. The resulting libraries were sequenced on an Illumina MiSeq with V2 chemistry on a 2,250 paired-end runs at the STRI molecular laboratory (Chavarria, 2021). A more in depth look at bacterial communities from this sequencing effort for the Agua Salud sites can be found in Chavarria et al. (2021).

### Data analysis

Statistical tests for total coliforms and *E. coli* data were performed on transformed log values following (Chavarria, 2021). One-half of the lower detection limit was substituted for values below the detection limit, and the upper detection limit was substituted for values above the maximum limit. Untransformed data were used for physicochemical parameters. All tests of significance used the non-parametric Kruskal-Wallis test with Benjamini–Hochberg corrections, as implemented in R v3.6.0 and the Coin package (R Core Team, 2012) (Table S1). Values were considered significant at *p* <0.05 (Chavarria, 2021).

The QIIME2 platform v2022.4 was used for the majority of the analysis of paired-end sequences, as described in Chavarria et al. (2021). Briefly, preprocessing consisting of primer trimming, sequence quality control, and forward and reverse read merging using QIIME2’s Cutadapt and DADA2 plugins to produce amplicon sequence variants (ASVs) (Bolyen et al., 2019; Callahan et al., 2016; Martin, 2011). Taxonomy was assigned using a naïve Bayesian classifier trained on the SILVA 99% sequence similarity database (v132) (Pruesse et al., 2007; Wang et al., 2007) and ASVs classified as mitochondria or chloroplast were removed from further analysis. The sequencing dataset was further filtered to only include ASVs assigned to genera classified as coliforms and genera that have previously been identified to cause false positives in culture-based indicator tests for total coliform and *E. coli* (Chao, 2006; Pisciotta et al., 2002). Genera classified and grouped as coliforms include *Escherichia*, *Citrobacter*, *Enterobacter*, *Hafnia*, *Serratia*, *Klebsiella*, and *Yersinia* (Leclerc et al., 2001; Yoon et al., 2013).

## Results

### Physicochemical parameters

Between 2018 and 2019, 21 streams were monitored and regularly sampled which resulted in a total of 315 samples collected, with a distribution of 13–19 samples per site. Water quality based on physicochemical parameters varied across land uses and sites (Table 1). Notably, temperature was found to be significantly different across land uses, with overall temperatures close to one degree Celsius higher in CP and SP sites than forested sites (Table 1, Table S1). pH was significantly higher in forested sites (MF and SF) than sites with cattle present (SP and CP). Turbidity was significantly higher in CP sites than forested sites (Table S1). Highest conductivity and total dissolved solids (Table S1) measurements were found in MF sites, while the lowest oxygen concentrations were found in SP and CP sites, although they were not significantly different from forested sites (Table 1, Table S1).

**Table 1:** Water quality metrics measured in 21 streams surrounded by four different land uses in the Panama Canal Watershed, Panama.

**Land Use**	**Site**	**Temperature**	**Turbidity**	**pH**	**Conductivity**	**Total Dissolved Solids**	**Dissolved Oxygen**	**Hardness**
		**Mean ± SD**	**Mean ± SD**	**Mean ± SD**	**Mean ± SD**	**Mean ± SD**	**Mean ± SD**	**Mean ± SD**
**Mature Forest**	SF-W1	25.3 ± 0.5	2.4 ± 1.8	6.2 ± 1.0	0.19 ± 0.15	84.0 ± 35.7	5.7 ± 1.4	25.0 ± 0.0
	SF-W2	24.9 ± 1.0	3.9 ± 3.6	6.7 ± 0.8	0.20 ± 0.09	95.8 ± 35.2	6.8 ± 1.0	31.3 ± 11.6
	SF-W3	25.3 ± 0.7	2.1 ± 1.4	6.8 ± 0.2	0.39 ± 0.19	118.2 ± 0.7	6.3 ± 1.3	40.5 ± 28.8
	SF-AS	25.1 ± 1.0	4.1 ± 4.3	7.0 ± 0.4	0.22 ± 0.05	132.6 ± 34.3	6.3 ± 4.3	94.2 ± 66.4
**Secondary Forest**	SF-W1	26.1 ± 0.9	5.3 ± 1.3	6.8 ± 0.6	0.18 ± 0.09	81.1 ± 22.0	5.3 ± 1.3	30.8 ± 11.0
	SF-W2	25.9 ± 1.6	21.4 ± 18	6.9 ± 0.5	0.18 ± 0.10	90.9 ± 33.9	6.9 ± 0.5	28.1 ± 10.1
	SF-W3	25.6 ± 1.0	4.6 ± 4.2	6.5 ± 0.6	0.22 ± 0.11	86.6 ± 18.1	5.9 ± 1.0	34.6 ± 12.7
	SF-AS	25.2 ± 0.9	1.5 ± 0.7	7.1 ± 0.3	0.13 ± 0.05	83.8 ± 14.2	6.0 ± 1.2	57.9 ± 31.1
**Silvopasture**	SP-W1	26.4 ± 1.7	9.0 ± 8.1	6.2 ± 0.8	0.14 ± 0.07	69.2 ± 18.2	5.2 ± 1.8	25.0 ± 0.0
	SP-W2	28.7 ± 3.1	13.8 ± 16.4	6.0 ± 0.8	0.15 ± 0.13	62.1 ± 26.8	5.8 ± 1.4	27.3 ± 7.5
	SP-W3	25.7 ± 1.2	7.5 ± 9.5	6.3 ± 0.6	0.26 ± 0.17	101.8 ± 31.4	5.4 ± 0.7	51.7 ± 23.6
	SP-W4	25.8 ± 0.8	20.6 ± 9.4	6.6 ± 0.2	0.30 ± 0.16	125.8 ± 55.3	6.4 ± 1.0	35.9 ± 28.9
	SP-AS	25.3 ± 0.9	1.9 ± 1.2	7.0 ± 0.3	0.14 ± 0.05	79.5 ± 14.0	5.0 ± 1.6	45.6 ± 9.8
**Cattle Pasture**	CP-W1	26.7 ± 0.8	4.4 ± 4.6	6.7 ± 0.6	0.20 ± 0.18	84.9 ± 21.8	5.8 ± 1.2	34.6 ± 12.7
	CP-W2	26.4 ± 1.0	2.6 ± 2.2	6.6 ± 0.7	0.18 ± 0.09	84.5 ± 18.9	5.7 ± 1.1	45.5 ± 10.1
	CP-W3	26.8 ± 1.9	4.4 ± 5.9	6.2 ± 0.8	0.18 ± 0.16	68.4 ± 33.7	6.1 ± 0.8	23.3 ± 6.5
	CP-W4	26.7 ± 1.5	20.1 ± 17.5	6.0 ± 0.8	0.17 ± 0.19	53.9 ± 26.7	5.0 ± 1.9	24.9 ± 9.8
	CP-W5	26.5 ± 1.5	11.8 ± 12.3	6.2 ± 0.7	0.21 ± 0.12	97.8 ± 44.8	5.2 ± 1.0	37.9 ± 16.0
	CP-W6	27.1 ± 1.5	29.8 ± 19.2	6.7 ± 0.6	0.24 ± 0.17	94.5 ± 22.4	6.7 ± 0.5	34.1 ± 13.2
	CP-W7	27.0 ± 1.8	2.1 ± 3.0	5.2 ± 1.4	0.27 ± 0.15	103.5 ± 23.0	6.4 ± 1.2	27.5 ± 7.9
	CP-AS	25.5 ± 1.1	16.4 ± 31.0	6.9 ± 0.5	0.19 ± 0.05	122.1 ± 40.9	5.0 ± 1.9	44.4 ± 10.7

**Notes.**

Values are mean ± standard deviation of measurements for each site over the one year sampling period.

MF Mature Forests (*n* = 68) SF Secondary Forests (*n* = 73) SP Silvopastures (*n* = 82) CP Cattle Pastures (*n* = 146)

### Coliform indicators and seasonal changes

**Figure 2:**
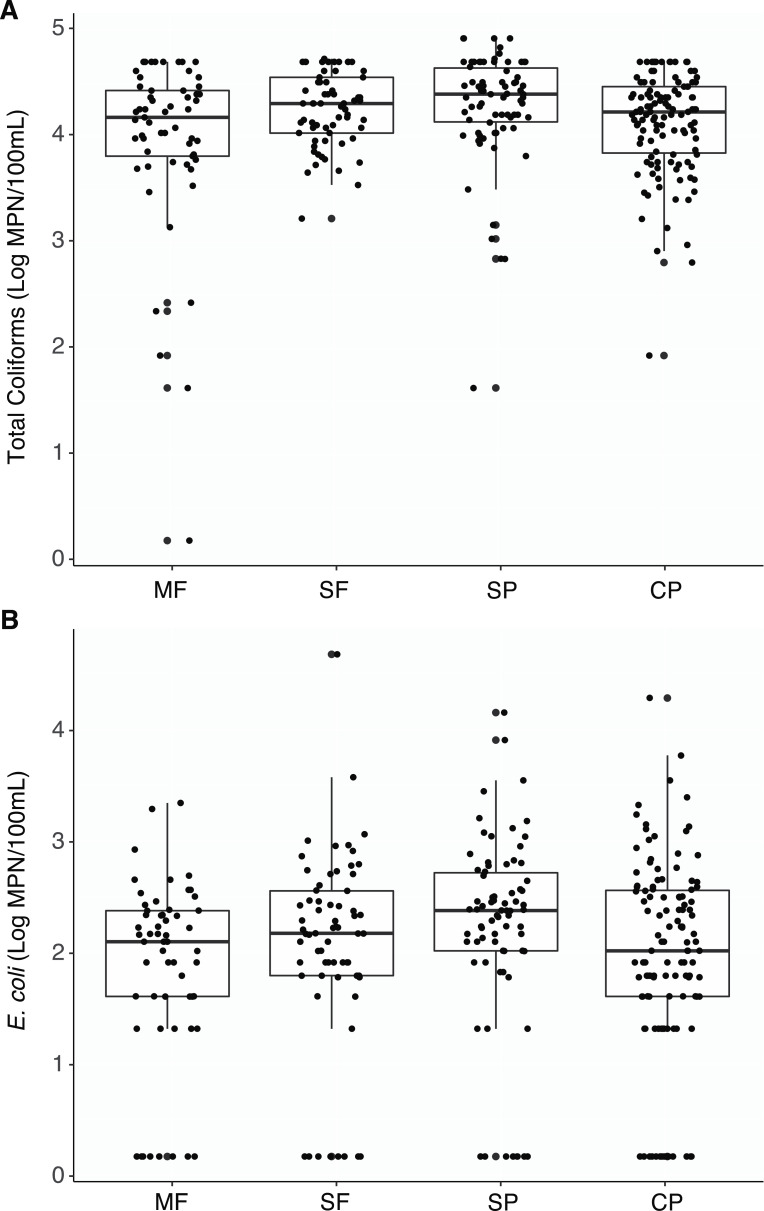
Culture-based indicator bacteria. Boxplots representing medians, quartiles, lower and upper extremes, and individual concentration of (A) Total coliforms and (B) *Escherichia coli* found across land uses. Concentrations were measured with the culture- based Colilert-18 IDEXX test and determined by the most probable number (MPN) statistical test. MF, Mature Forests (*n* = 68); SF, Secondary Forests (*n* = 73); SP, Silvopastures (*n* = 82); CP, Cattle Pastures (*n* = 146).

Total coliforms were found to be ubiquitous across all sites and there were no significant differences in concentrations between the different land uses when aggregating all measurements by the different land-use types (Table S1, Fig. 2A). Only two samples had undetectable levels of coliform bacteria (two MF samples). While some differences between sites within a land use and seasons within sites were observed, the majority of these comparisons were not significant (Figs. S1 and S2).

*Escherichia coli* was also detected at all sites and no significant differences were found between land use types (Table S1, Fig. 2B). Eighty seven percent of samples had measurable concentrations of *E. coli* (274/315 samples) and undetectable concentrations within each land use was below 15% (MF = 14.0%, SF = 12.3%, SP = 10.8%, CP = 14.3%). However, concentrations were more variable, and no seasonal shifts were found; only a few differences in concentrations were observed between the dry and wet seasons at sites where cattle were present (Fig. S3).

### Identification of coliform bacteria with high-throughput sequencing

**Figure 3:**
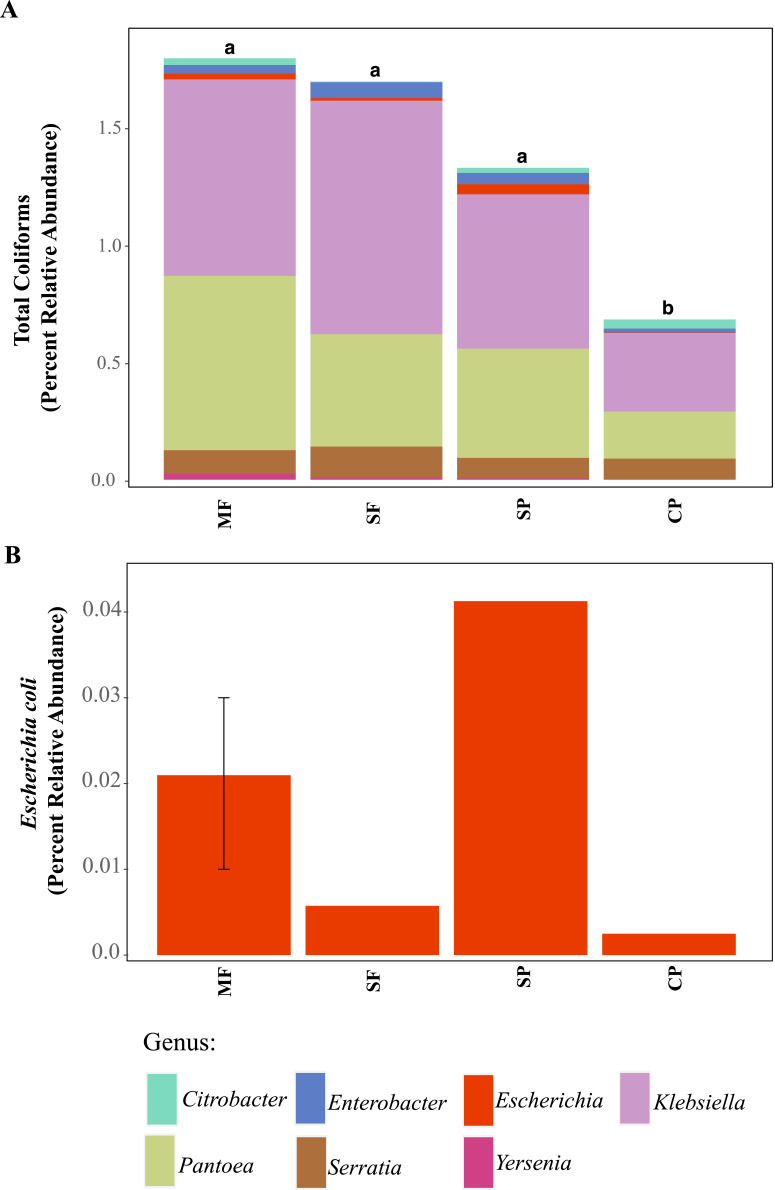
ASVs assigned to indicator bacteria. Relative abundance of ASVs assigned to (A) genera that are classified as coliforms and (B) *Escherichia coli* using 16S rDNA metabarcoding. MF, Mature Forests (*n* = 68); SF, Secondary Forests (*n* = 73); SP, Silvopastures (*n* = 82); CP, Cattle Pastures (*n* = 146). Relative abundances were calculated from samples rarefied to 2200 sequences per sample. Letters indicate significant differences between land uses (*p* < 0.05) based on Kruskal-Wallis and pairwise Wilcox (with Benjamini- Hochberg correction) tests.

Using 16S rDNA metabarcoding, we detected several genera that are classified as total coliforms and found that their relative abundances were more variable across land uses, sites, and seasons than seen in our culture-based tests results (Fig. 3A and Fig. S4). Total coliform represented less than 2% of the bacterial communities across all sites (for full Agua Salud sites data please see Chavarria et al., 2021) and coliform bacteria abundance was significantly lower in CP sites than other land uses (Table S1, Fig. 3A). As coliform bacteria are known to be ubiquitous in the environment and their presence alone does not signify fecal contamination in water environments (McMath et al., 1999; Somaratne & Hallas, 2015), it was not surprising to detect them across all land uses. Although the relative abundance of coliform bacteria was not significantly different across MF, SF, and SP (for Agua Salud sites), significant differences were found in the overall bacterial communities across these land-use sites (Chavarria et al., 2021). However, metabarcoding detected *E. coli* at only five of our 21 sampling sites (seven samples in total: MF = 3, SF =1, SP = 2, CP = 1) and where it was found, it represented only a small proportion of the total coliform community (Figs. 3A and 3B).

**Figure 4:**
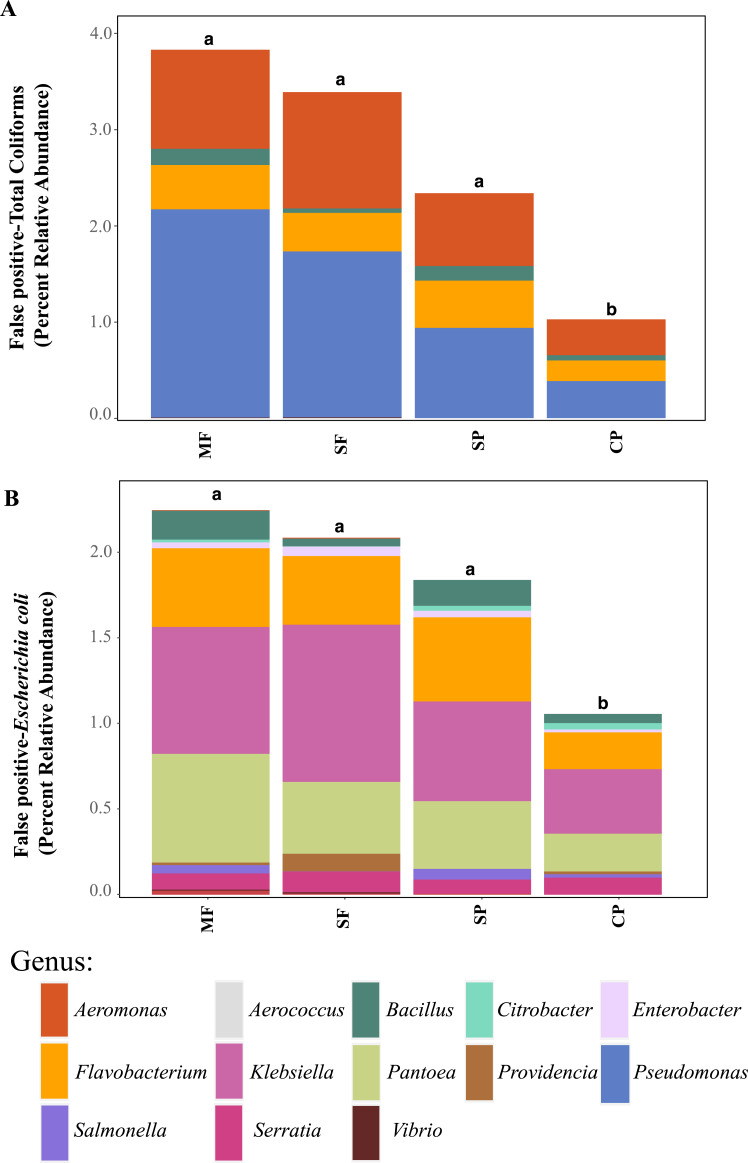
Potential false-positives in common culture-based indicator bacteria tests. Relative abundance of ASVs assigned, through 16S rDNA metabarcoding, to genera known to produce false-positives in common culture-based indicator tests measuring (A) Total coliforms and (B) *Escherichia coli* (Pisciotta et al., 2002; Chao, 2006). MF, Mature Forests (*n* = 68); SF, Secondary Forests (*n* = 73); SP, Silvopastures (*n* = 82); CP, Cattle Pastures (*n* = 146). Relative abundances were calculated from samples rarefied to 2200 sequences per sample. Letters indicate significant differences between land uses (*p* < 0.05) based on Kruskal-Wallis and pairwise Wilcox (with Benjamini–Hochberg correction) tests.

To better understand why Colilert^®^ tests results showed high concentrations with no differences across land uses while metabarcoding detected variable abundances of total coliform and few samples containing *E. coli*, we did further analysis of ASVs assigned to taxa that have been reported to cause false positive results in culture-based tests like Colilert^®^ that are based on the enzymatic properties *β*-galactosidase (total coliforms) and *β*-glucuronidase (*E.coli*) (Maheux et al., 2015). *Aeromonas* (Gammaproteobacteria) and *Pseudomonas* (Gammaproteobacteria) were among the most abundant genera capable of producing false positives results in total coliforms (Fig. 4A, Fig. S5) and are both known to be ubiquitous in aquatic systems (Usui et al., 2016; Valério, Chaves & Tenreiro, 2010). *Flavobacterium* (Bacteroidetes), *Klebsiella* (Gammaproteobacteria), *Pantoea* (Gammaproteobacteria), and *Serratia* (Gammaproteobacteria) were the most abundant genera capable of causing false positives for *E. coli* (Fig. 4B, Fig. S6). These genera are common in soil, water and associated plants (Bernardet & Bowman, 2006; Johnson et al., 2007; Lopez-Torres, Hazen & Toranzos, 1987; Thompson et al., 2007). All sites showed presence of one or more of these genera with higher relative abundances in mature forest sites (Fig. S6, Table S1), creating multiple opportunities for false positives in the Colilert^®^ test. Thus, non-target bacteria that are part of the natural ecology of these tropical oligotrophic water environments and their surroundings may confound traditional FIB culture-based water quality indicators in these environments.

**Figure 5:**
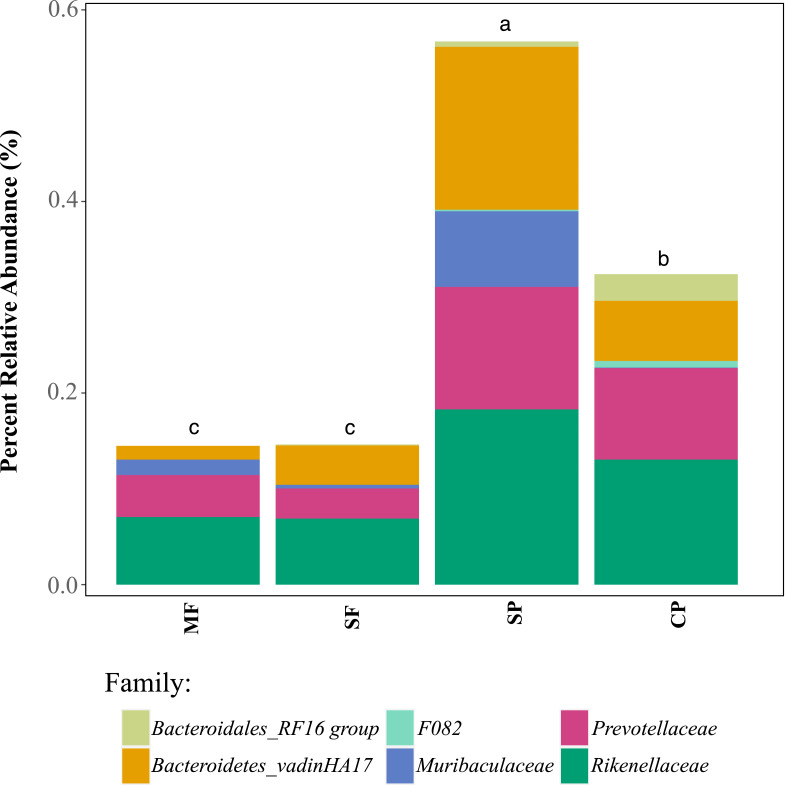
Relative abundance of families in the order Bacteroidales that are known to occur in livestock fecal material, as determined with 16S metabarcoding. MF, Mature Forests (*n* = 68); SF, Secondary Forests (*n* = 73); SP, Silvopastures (*n* = 82); CP, Cattle Pastures (*n* = 146). Relative abundances were calculated from samples rarefied to 2200 sequences per sample. Letters indicate significant differences between land uses (*p* < 0.05) based on Kruskal-Wallis and pairwise Wilcox (with Benjamini–Hochberg correction) tests.

To further assess the potential for fecal contamination, we searched for sequences that matched other bacterial members known to be part of the intestinal tract of warm-blooded animals and common fecal contaminants. We found several families in the order Bacteroidales with relative abundances that were two times higher in streams influenced by pastures than forested streams (Fig. 5). Bacteroidales (phylum Bacteroidetes) are commonly found in the feces of humans and other warm-blooded animals and their presence in water is an indication of fecal pollution and the possible presence of enteric pathogens (Ridley et al., 2014; Tambalo et al., 2012).

## Discussion

Natural streams provide an important water source for human settlements in rural areas across the tropics. Within the last decades, many natural landscapes have undergone significant changes due to anthropogenic activity and climate change. This in turn has significantly affected surface waters and other water environments. In this study, coliform bacteria in 21 oligotrophic tropical streams influenced by four common land uses in the PCW were investigated. These streams were selected due to their location, preservation, and lack of upstream land influences that could confound our results. Although considerable resources are expended annually to measure water quality of drinking water sources (both groundwater and surface waters, such as lakes and rivers), a better understanding of the effectiveness of water quality measurement techniques is needed to improve the efficiency of water resource management. We evaluated water quality across four common land use scenarios in central Panama and hypothesized that streams that were influenced by cattle would show lower water quality than streams in undisturbed mature forest catchments. Based on our chemical and physical measurements, water quality across the four land uses in this study was consistent with previous studies (Ogden et al., 2013; Uriarte et al., 2011; Yu et al., 2013), and streams where cattle were present (CF and SF) had higher temperatures, higher turbidities, and lower dissolved oxygen than forested sites (SF and MF). Thus, we expected culture-based tests to also reflect the different water qualities found across land uses and sites. However, using the culture-based Colilert^®^ test, total coliforms and *E. coli* were found to be ubiquitous across all sites, with no significant differences between forested sites and sites with cattle present. The highest concentrations of total coliforms were found in SP sites (10^4^ MPN/100 mL), but all land uses had measurable concentrations of coliform bacteria throughout the study that reached similar orders of magnitude. These results contradict our original hypothesis that streams influenced by cattle pastures would result in higher prevalence of FIB than streams adjacent to forests.

Previous studies have alluded to the potential drawbacks of using common culture-based indicator bacteria tests, that were designed to test for *E.coli* in drinking water or surface water samples in temperate regions, to assess water quality in tropical environments (Eccles et al., 2004). Chao (2006) analyzed fresh water samples from nine tropical rivers in Taiwan and found that false positive rates for *E.coli* and coliforms using Colilert^®^ were 36.4% and 10.3%, respectively. Pisciotta et al. (2002) also found false positive rates for *E.coli* to be 27.3% when assessing Florida marine waters. These studies, and others, have also raised questions regarding the use of culture-based tests and the reliability of indicator bacteria such as *E.coli* to assess microbial water quality due to their poor correlation with pathogens (Ahmed, Goonetilleke & Gardner, 2010; Goh et al., 2019; Luyt et al., 2012; Peperzak & Bleijswijk, 2020; Sercu et al., 2011; Shibata et al., 2004).

Despite this, culture-based tests that depend on selective media, such as Colilert^®^ that depends on the enzymatic properties *β*-galactosidase and *β*-glucuronidase, continue to be a widely used approach to study and monitor water quality in different environments (Berendes et al., 2018; Chuah & Ziegler, 2018; Khairunnisa, Hendrawan & Fachrul, 2021; Morgan, Opio & Migabo, 2020; Saeidi et al., 2018; Tsuchioka et al., 2019; Tyner et al., 2018) as they are easy to use, affordable, and standardized. These advantages are not small, especially in resource-constrained environments where the monitoring of water quality might be done by personnel with minimal training. These tests can be very effective at detecting contamination in some water environments, such as drinking water systems. They can show differences between locations and even seasonal variability in temperate environments (Bain et al., 2015; Rao et al., 2015; Vital et al., 2017, this study); However, the detection of coliforms in tropical freshwater environments may not indicate fecal contamination and we caution their use in certain tropical settings, such as natural stream environments with no direct sources of pollution (Rochelle-Newall et al., 2015). Overestimation of *E. coli* in these systems may have large implications for both small landholders and large municipalities who depend on surface waters for their livelihoods, such as for drinking water and irrigation purposes. In Panama, regulations for total coliforms in recreational waters is <250 colony forming units or MPN/100mL (COPANIT, 2019; MEF, 2008), concentrations that are orders of magnitude lower than the ones found in this study. Source-water protection programs that integrate superior approaches to monitoring water quality and water microbiomes are key for the protection of watershed characteristics, water quality and public health.

Our metabarcoding revealed that coliform bacteria was a minor fraction of the total bacterial community present. Less than four percent of total bacterial relative abundance was assigned to total coliform members and sequences assigned to *E. coli* were found in only seven samples in five sites. Additional studies increasing sequencing depth or using *E. coli* specific amplification primers and qPCR are needed to resolve the detection limitations of the metabarcoding technique when assessing water quality. In addition, although we were unable to sequence the cultures of total coliform-positive Colilert^®^ quantitrays, future work sequencing positive Colilert^®^ tests is needed to determine which bacteria are present and to quantify the incidence of false positive results.

However, 16S metabarcoding can be a useful tool to understand microbial community changes and guide source-tracking efforts. For example, our data suggests that other taxa, such as member of the order Bacteroidales, could be better indicators of fecal contamination in tropical freshwater habitats than *E. coli*. Alternative molecular assays with Bacteroidales targets, like the universal Bacteroidales Taqman^®^ assay BacUni-UCD or the livestock-associated BacCow test, have been developed and shown to be effective at identifying non-human sources of fecal pollution (Holcomb & Stewart, 2020; Jenkins et al., 2009; Teixeira et al., 2020). In natural systems in the tropics, testing for alternative taxa, such as Bacteroidales, may be a more accurate way to identify actual fecal pollution in watersheds and other water systems. Ultimately, a combined approach of multiple methods may be needed and further research is needed to develop a comprehensive approach to effectively assess fecal pollution in tropical environments.

## Conclusions

• Measurable concentrations of both total coliforms and *E.coli* in the water column were found in samples from all sites using a traditional culture-based protocol (Colilert^®^) and there were no significant differences between total coliform or *E.coli* concentrations across different land use types, including mature forest and active cattle pastures.
• 16S rDNA metabarcoding showed variability between land uses in the relative abundance of genera recognized as coliforms but did not consistently detect *E. coli* across sites.
• 16S rDNA metabarcoding identified various genera across all samples which could cause false positives in culture-based Colilert^®^ tests.
• Members of the order Bacteroidales were significantly more abundant in sites with cattle present, suggesting their utility as indicators of fecal contamination.
• Common culture-based indicator bacteria tests targeting coliforms might not be appropriate for assessing fecal contamination in tropical oligotrophic stream environments.

##  Supplemental Information

10.7717/peerj.18007/supp-1 Supplemental Information 1 Water quality metrics measured in 21 streams

## Additional Information and Declarations

### DNA Deposition

The following information was supplied regarding the deposition of DNA sequences: The raw 16S rDNA sequences are available at NCBI: PRJNA918336.

### Data Availability

The following information was supplied regarding data availability: The raw data is available at NCBI PRJNA918336 and at Chavarria, Karina (2023): AS-Raw-Data-Indicator-bacteria-project.csv. Smithsonian Tropical Research Institute. Dataset. https://doi.org/10.25573/data.21225338.
https://www.ncbi.nlm.nih.gov/bioproject/?term=PRJNA918336
The site raw data is available at figshare: Chavarria, Karina; Saltonstall, Kristin; Hall, Jefferson (2024). 16S rDNA metabarcoding raw data files - Forested sites 1. Smithsonian Tropical Research Institute. Dataset. https://doi.org/10.25573/data.21445482.v1
Chavarria, Karina; Saltonstall, Kristin; Hall, Jefferson (2024). 16S rDNA metabarcoding raw data files - Pasture sites 1. Smithsonian Tropical Research Institute. Dataset. https://doi.org/10.25573/data.21445650.v1.
