# Supplementary material for: Widespread occurrence of fecal indicator bacteria in oligotrophic tropical streams. Are common culture-based coliform tests appropriate?
Source: PeerJ. 2024 Sep 6;12:e18007. doi: 10.7717/peerj.18007 (PMC11382651; doi:10.7717/peerj.18007)
Supplement: Supplemental Information 1 [file peerj-12-18007-s001.docx]

Supplemental Information

**Widespread occurrence of fecal indicator bacteria in oligotrophic tropical streams. Are common culture-based coliform tests appropriate?**

*Karina A. Chavarria^1,2*^, Jorge Batista^1^,* and *Kristin Saltonstall^1^*

*^1^* Smithsonian Tropical Research Institute (STRI), Amador, Naos, Panama

^2^ Department of Civil and Environmental Engineering, University of Massachusetts Amherst, Amherst, Massachusetts, United States of America

Corresponding Author: Karina A. Chavarria

Smithsonian Tropical Research Institute

Amador, Naos, Panama

Chavarria.karina.a@gmail.com

Supplemental Figure 1-6

Supplemental Table 1

**Supplemental Figure 1**. **Culture-based indicator bacteria concentrations.** Boxplots representing medians, quartiles, lower and upper extremes, and individual concentration of total coliforms and *E. coli* found across each site in this study. Concentrations were measured with the culture-based Colilert-18 IDEXX test and determined by the most probable number (MPN) statistical test. MF = Mature Forests (n=68); SF = Secondary Forests (n=73); SP = Silvopastures (n=82); CP = Cattle Pastures (n=146).

**Supplemental Figure 2**. **Total coliform concentrations (mean ± SD) during the dry and wet seasons (2019) at 21 sampling sites in the Panama Canal Watershed**. Bars represent the mean concentration measured by the Most Probable Number (MPN, Oblinger & Koburger; 1975). Asterix indicates significant differences between seasons within a site. Significance was determined based on Kruskal-Wallis and pairwise Wilcox (with Benjamini-Hochberg correction) tests. MF = Mature Forests (n=68); SF = Secondary Forests (n=73); SP = Silvopastures (n=82); CP = Cattle Pastures (n=146).

**Supplemental Figure 3**. ***Escherichia coli* concentrations (mean ± SD) during the dry and wet seasons (2019) at 21 sampling sites in the Panama Canal Watershed**. Bars represent the mean concentration measured by the Most Probable Number (MPN, Oblinger & Koburger; 1975). Asterix indicates significant differences between seasons within a site. Significance was determined based on Kruskal-Wallis and pairwise Wilcox (with Benjamini-Hochberg correction) tests. MF = Mature Forests (n=68); SF = Secondary Forests (n=73); SP = Silvopastures (n=82); CP = Cattle Pastures (n=146).

**Supplemental Figure 4**. **ASVs assigned to total coliforms by season during 2018-2019**. Relative abundance of ASVs assigned to genera that are classified as Coliforms through 16S rDNA metabarcoding. Samples collected over the year 2019. MF = Mature Forests (n=68); SF = Secondary Forests (n=73); SP = Silvopastures (n=82); CP = Cattle Pastures (n=146).

Relative abundances were determined from samples rarefied to 2200 sequences per sample.

**Supplemental Figure 5**. Relative abundances of **ASVs assigned to genera that have been found to cause false-positives in culture-based tests for total coliforms by season for each site.** Samples were collected in 2019. MF = Mature Forests (n=68); SF = Secondary Forests (n=73); SP = Silvopastures (n=82); CP = Cattle Pastures (n=146). Relative abundances were determined from samples rarefied to 2200 sequences per sample.

**Supplemental Figure 6.** Relative abundances of **ASVs assigned to genera that have been found to cause false-positives in culture-based tests for *Escherichia coli* by season for each site.** Samples were collected in 2019. MF = Mature Forests (n=68); SF = Secondary Forests (n=73); SP = Silvopastures (n=82); CP = Cattle Pastures (n=146). Relative abundances were determined from samples rarefied to 2200 sequences per sample.

**Supplemental Table 1. Physiochemical parameters and bacterial indicator comparison across land uses.** Kruskal-Wallis and pairwise Wilcox (with Benjamini-Hochberg correction) tests were used to determine significance. MF = Mature Forests (n=68); SF = Secondary Forests (n=73); SP = Silvopastures (n=82); CP = Cattle Pastures (n=146).
